# Low β predicts motor output and cell degeneration in the A53T Parkinson’s disease rat model

**DOI:** 10.1093/brain/awaf063

**Published:** 2025-02-18

**Authors:** Katarina Hofman, Jiazhi Chen, Tanmoy Sil, Franziska Pellegrini, Stefan Haufe, Nicolo Pozzi, Chiara Palmisano, Ioannis U Isaias, James B Koprich, Jonathan M Brotchie, Andrea A Kühn, Cordula Matthies, Martin M Reich, Muthuraman Muthuraman, Jens Volkmann, Chi Wang Ip

**Affiliations:** Department of Neurology, University Hospital of Würzburg, 97080 Würzburg, Germany; Department of Neurology, University Hospital of Würzburg, 97080 Würzburg, Germany; Department of Neurology, University Hospital of Würzburg, 97080 Würzburg, Germany; Uncertainty, Inverse Modeling and Machine Learning Group, Technische Universität Berlin, 10623 Berlin, Germany; Uncertainty, Inverse Modeling and Machine Learning Group, Technische Universität Berlin, 10623 Berlin, Germany; Department of Neurology, University Hospital of Würzburg, 97080 Würzburg, Germany; Department of Neurology, University Hospital of Würzburg, 97080 Würzburg, Germany; Department of Neurology, University Hospital of Würzburg, 97080 Würzburg, Germany; Atuka Inc., Toronto, Ontario, Canada M5X 1C9; Atuka Inc., Toronto, Ontario, Canada M5X 1C9; Department of Neurology, Movement Disorder and Neuromodulation Unit, Charité—Universitätsmedizin, 10117 Berlin, Germany; Department of Neurosurgery, University Hospital of Würzburg, 97080 Würzburg, Germany; Department of Neurology, University Hospital of Würzburg, 97080 Würzburg, Germany; Department of Neurology, University Hospital of Würzburg, 97080 Würzburg, Germany; Informatics for Medical Technology, Institute of Computer Science, University Augsburg, 86159 Augsburg, Germany; Department of Neurology, University Hospital of Würzburg, 97080 Würzburg, Germany; Department of Neurology, University Hospital of Würzburg, 97080 Würzburg, Germany

**Keywords:** local field potentials, β burst dynamics, α-synuclein, structural equation modeling, neurodegeneration

## Abstract

Elevated β (13–30 Hz) synchronization within the subthalamic nucleus (STN) characterizes bradykinesia in Parkinson’s disease (PD). β oscillations may serve as biomarkers for off-period motor symptoms and control signals for adaptive, closed-loop deep brain stimulation (DBS) in PD. However, their relation to striatal dopaminergic denervation and PD progression remains uncertain. Research on β oscillations is limited to advanced PD stages undergoing DBS, prohibiting insights into early-stage progression and compensatory mechanisms. We therefore investigated β dynamics, correlation with motor performance, and nigrostriatal neurodegeneration in a progressive PD rat model overexpressing AAV1/2-A53T α-synuclein, mimicking PD pathology.

Over 8 weeks, we longitudinally conducted behavioural assessments using the cylinder test and recorded local field potentials (LFP) from the STN and motor cortex (MCx) in the AAV-A53T-αSyn PD rat model.

Increased β power and burst parameters accompanied early motor deficits in the AAV-A53T-αSyn PD rat model. Changes were observed in the STN and MCx versus empty vector controls; alterations intensified with pathology progression. Increased high β power and burst parameters (e.g. long burst probability in the STN but not MCx) were associated with motor impairment and nigrostriatal dopaminergic neurodegeneration. Multivariate analyses from these rat-derived data demonstrated that combined β parameters in the cortico-subthalamic pathway and striatal dopaminergic fibre density predicted motor performance and neurodegeneration. Additional multivariate analyses confirmed the translational relevance of the A53T PD model, linking β activity and dopamine uptake to motor impairment (UPDRS III Med-OFF) in human PD patients. Our data support the pathophysiological significance of β oscillations as a progression marker of PD for motor symptoms and neurodegeneration. Our predictive models carry translational relevance, with the prospect of monitoring disease progression and neuroprotective outcomes in PD based on LFP recordings.

## Teaser

By recording from the subthalamic nucleus and motor cortex of a Parkinson’s disease rat model, Hofman *et al.* show that beta oscillations can function as a progression marker for motor symptoms and neurodegeneration. These findings have potential translational relevance for assessing outcomes of neuroprotective interventions.

## Introduction

Parkinson’s disease (PD) is characterized by a progressive, chronic loss of dopaminergic neurons and striatal denervation, resulting in motor symptoms such as akinesia, rigidity and rest tremor. A strong association between motor impairment and heightened synchronization of oscillatory neural networks in the β range (13–30 Hz) has been observed in both the basal ganglia and motor cortex (MCx) of PD patients and animal models, which closely aligned with symptom severity.^1-4^ Notably, pathologically increased β power in PD, as determined by analysis of local field potentials (LFPs), was significantly attenuated by effective symptomatic interventions such as levodopa intake or subthalamic nucleus (STN) deep brain stimulation (DBS).^4-10^ However, although attention has increasingly turned towards understanding the role of pathological LFPs in PD pathophysiology, the precise interrelation between these aberrant LFP patterns, the degeneration of dopaminergic neurons within the substantia nigra (SN) and the denervation of dopaminergic terminals in the striatum is unknown. Moreover, while these β-band oscillations are considered a potential state marker for off-period motor symptoms that could be used as control signals for adaptive, closed-loop DBS in PD,^11-14^ when β oscillations evolve within the progressive course of PD and how they relate to symptom expression and dopaminergic neurodegeneration remains elusive.

The main hypothesis underlying the present work is that β oscillations recorded from STN and MCx serve as a pathophysiological biomarker of progressive network dysfunction in PD, which is strongly interrelated to the course of motor impairment and dopaminergic neurodegeneration.

As clinical LFP recordings are typically limited to patients with advanced PD undergoing DBS, we turned to a rat model of PD that exhibits motor impairment, progressive nigrostriatal degeneration and Lewy-like pathology—closely mimicking human PD^15^—to address our main hypothesis. Specifically, we aimed (i) to characterize β oscillatory dynamics in both STN and MCx during the early and progressive stages of PD using the AAV-A53T-α-synuclein PD rat model (hαSyn) that is generated by injecting an AAV1/2 virus expressing the human mutated A53T α-syn into the SN of wild-type rats; (ii) to investigate the association between β power and burst parameters with motor deficits and nigrostriatal dopaminergic degeneration; and (iii) to evaluate whether combined β parameters in the cortico-subthalamic pathway and striatal dopaminergic fibre density can predict motor performance and neurodegeneration, thereby establishing β oscillations as a reliable biomarker for PD progression.

## Materials and methods

### Animals

**Figure 1:**
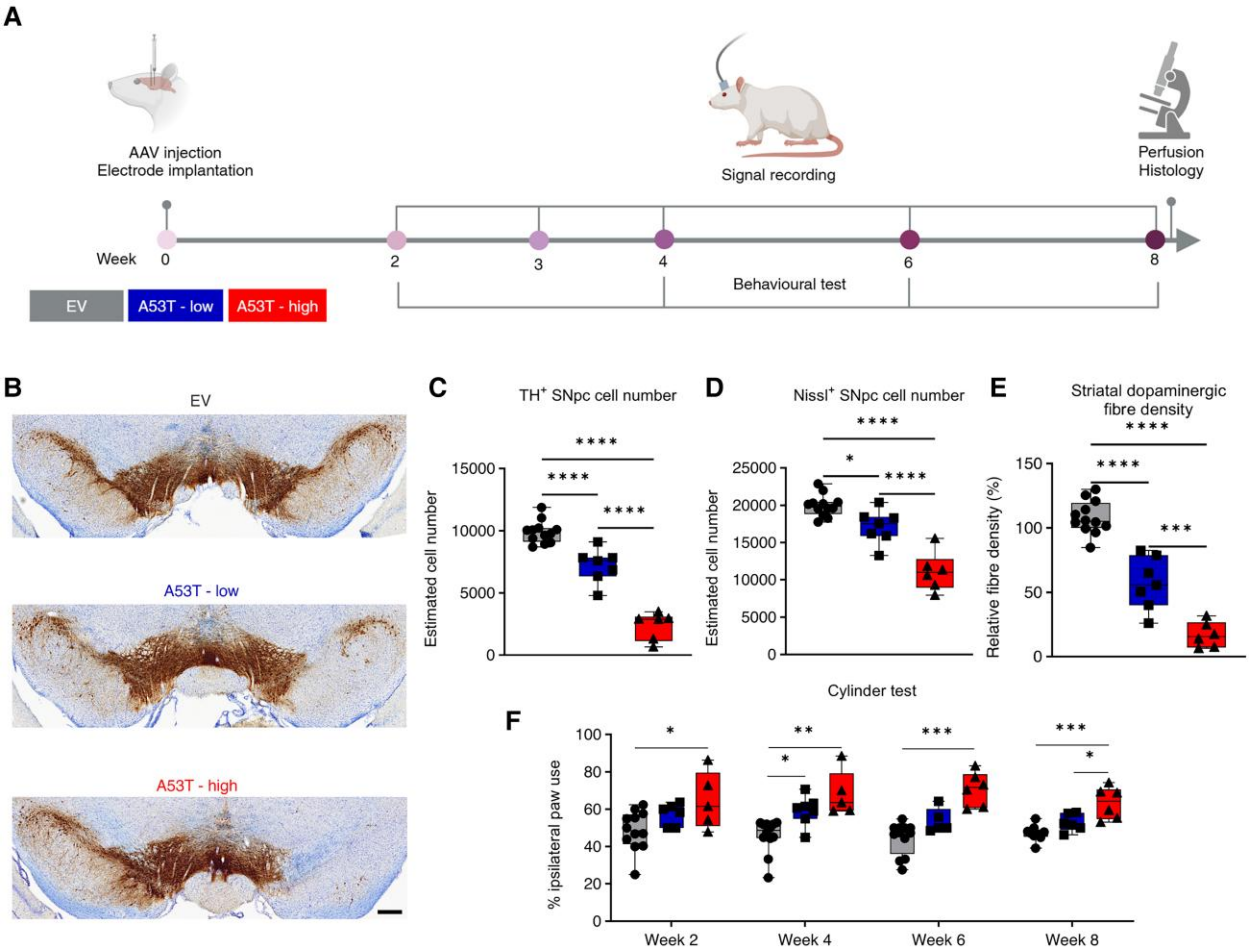
**AAV-dose-dependent dopaminergic neurodegeneration and motor impairment in the AAV-A53T-αSyn Parkinson’s disease rat model**. (**A**) Experimental design over 8 weeks. Created in BioRender. Ip, A. (2025) https://BioRender.com/w20u214. (**B**) Representative images of SNpc TH^+^ Nissl double staining of EV, A53T-low and A53T-high rat SNpc (scale bar: 400 μm). (**C**) Number of TH^+^ [*F*(2,22) = 95.20, *P* < 0.0001] and (**D**) Nissl^+^ [*F*(2,22) = 39.22, *P* < 0.0001] neurons in the SNpc and (**E**) TH^+^ fibre density in the striatum [*F*(2,22) = 81.70, *P* < 0.0001 of EV (grey; *n* = 12), A53T-low (blue; *n* = 7) and A53T-high (red; *n* = 6) rats. (**F**) Motor performance assessed by the cylinder test over an 8-week time frame [Week 2: *F*(2,20) = 4.125, *P* = 0.0316 (*n* = 12 for EV, *n* = 6 for A53T-low, *n* = 5 for A53T-high); Week 4: *X^2^* = 14.22, *P* = 0.0008 (*n* = 12 for EV, *n* = 7 for A53T-low, *n* = 5 for A53T-high); Week 6: *X^2^* = 14.59, *P* = 0.0007 (*n* = 12 for EV, *n* = 5 for A53T-low, *n* = 6 for A53T-high); Week 8: *F*(2,18) = 13.95, *P* = 0.0002 (*n* = 9 for EV, *n* = 6 for A53T-low, *n* = 6 for A53T-high)]. Data are presented as whiskers + min to max (**C**–**F**). *P < 0.05, **P < 0.01, ***P < 0.001 and ****P < 0.0001 for the *post hoc* comparisons of one-way ANOVA or Kruskal–Wallis test. EV = empty vector; SNpc = substantia nigra pars compacta; TH = tyrosine hydroxylase.

Twenty-five adult male Sprague-Dawley rats (Charles River Laboratories), weighing 250–310 g, were kept in a temperature- and humidity-controlled environment (21°C, 12-h light/dark cycle), with access to food and water *ad libitum.* All applicable international, national and/or institutional guidelines for the care and use of animals were followed. The local authorities at the Regierung von Unterfranken, Würzburg, Germany, approved all animal experiments under number 55.2.2–2532.2–767. The experimental design is depicted in Fig. 1A.

### Surgical procedure

In a single operative procedure, stereotaxic guidance was used to perform a unilateral AAV injection into the right SN alongside the implantation of chronic electrodes in the MCx and STN in the right hemisphere.^15^ Rats were anaesthetized with 1.5%–2.5% isoflurane and carprofen. Either 2.0 µl of empty AAV1/2 (empty vector; EV) or 2.0 µl of AAV1/2-expressing human mutated A53T-αSyn was delivered into the SN using the coordinates from bregma: −5.2 mm anteroposterior (A/P), −2.0 mm mediolateral (M/L), −7.4 mm dorsoventral (D/V).^16^ To achieve diverse levels of dopaminergic degeneration, we injected two different concentrations of AAV1/2-A53T-αSyn: 2.55 × 10^12^ (A53T-low) and 15.3 × 10^12^ (A53T-high) genomic particles (gp)/ml. An epidural screw electrode (1 mm diameter; Plastics One Inc.) for electrocorticogram (ECoG) electrophysiological recordings was implanted over the primary MCx (M1; coordinates: A/P +3.0 mm, M/L −3.0 mm). A single, stainless-steel perfluoroalkoxy-coated wire electrode (Science Products) was implanted into the ipsilateral STN (coordinates: A/P −3.6 mm, M/L −2.5 mm, D/V −7.7 mm). Both EcoG and LFP recording electrodes were referenced against an epidural screw electrode implanted over the cerebellum (A/P −9.0 mm, M/L +3.0 mm). Electrode pins were connected to a six-channel socket (Plastics One Inc.) and cemented with ultraviolet dental cement (Filtek Supreme XTE Composite, 3M) and dental acrylic cement (Paladur, Kulzer GmbH).

### Behavioural testing

To test for forelimb use asymmetry, we performed the cylinder test as previously described.^17^ Every rat was videotaped before surgery (baseline) and every second week over 8 weeks. Scoring for rearing and the number of touches to the cylinder wall was performed *post hoc* by an observer who was blinded to the treatment condition, as described.^18^ Results were presented as percentage of the ipsilateral (right) forepaw use by calculation, using the equation: [(ipsilateral paw + 0.5 both paws)/(ipsilateral paw + contralateral paw + both paws)] × 100. The calculated percentage indicates the preference of the forepaw use as follows: 50% = symmetric use of both forepaws; <50% = preference of the left forepaw; >50% preference of the right forepaw.^17^

### Electrophysiology

#### ECoG/LFP recordings

Following 2 weeks of post-surgical recovery, simultaneous ECoG and LFP recordings were obtained for 40 min during the spontaneous activity patterns. Subsequently, recording sessions were conducted every second week, with an additional recording session at Week 3. All recordings were performed at the same time, starting at 7 a.m. Recordings were conducted during the awake, resting state when the rats’ eyes were open. Signals were amplified (A-M System Inc. Model 3600), band-pass filtered (0.3–100 Hz) and digitized at a sampling frequency of 256 Hz (Spike 2, CED system). The recorded signals were then stored on a computer for further offline analysis.

#### Signal analysis

ECoG and LFP signals that were free of major artefacts were included for analysis. For each rat, three 30-s epochs were marked, based on the videotaped motor behaviour and signal characteristics from each recording session, ensuring they were artefact-free and occurred during a calm awake state with eyes open (no movement). All signal analyses were conducted using MATLAB (version 2019b, Mathworks, Natick, MA, USA) and utilized functions from Fieldtrip Toolbox19 and a custom-written MATLAB script. Power spectra were calculated using Fast Fourier Transformation with a Hanning window function, with 1-Hz resolution without overlapping. Absolute power values were normalized to the sum of total power of 5–45 and 55–95 Hz. The functional interactions between STN and MCx were estimated using imaginary coherence. This method calculates the imaginary part of coherency, reflecting true functional coupling while mitigating false connectivity from zero-lag interactions such as volume conduction.^20^ A 1-s window with zero overlap was employed for this calculation. In burst analyses, signals were normalized using *z*-score [(X–μ)/δ)] to avoid amplitude variability between subjects.^1^ We utilized a 10-cycle wavelet transform (Morlet wavelet, frequency range 1–128 Hz, resolution 1 Hz), computed the average of the mean signal within the target frequency band and calculated the absolute values of power for this frequency band. Oscillation periods above the 75th percentile of the power^9^ were recognized as β bursting with a minimal duration of 100 ms. The common amplitude threshold was determined for all MCx, and the other for all STN signals.^3^ Burst amplitude, rate and long burst (i.e. longer than 350 ms) probability were calculated for each group.

### Tissue processing and histology

After the final recording session, rats underwent transcardial perfusion with 0.1 M phosphate buffered saline (PBS). The brain’s rostral part, including the striatum, was snap-frozen in dry ice-cooled isopentane and cut into 10-µm coronal sections. The caudal part, containing the STN and SN, was post-fixed in 4% paraformaldehyde, cryoprotected in 30% sucrose/PBS, frozen in dry ice-cooled isopentane and cut into 40-µm coronal sections, collected in six series.

To measure the optical density (OD) of dopaminergic fibres in the striatum and analyse tyrosine hydroxylase (TH)+ cell numbers in the substantia nigra pars compacta (SNpc), both fresh-frozen 10-µm sections and free-floating 40-µm sections were immunostained using specific antibodies and visualization techniques. Staining protocols used for tissue analysis (TH, αSyn, DAPI and Nissl) have previously been described in detail.^15^ The OD of TH^+^ dopaminergic fibres in striatum was quantified using ImageJ, corrected for background density and normalized with the intact side’s average values, expressed as a percentage.^21^ Average values were obtained from three different anatomical levels of the striatum (anterior, commissural and posterior; Supplementary Fig. 1A).

### Unbiased stereological quantification of SNpc neurons

Stereological quantification of SNpc neurons was done using Stereo Investigator software (version 11.07; MicroBrightField Biosciences, Williston, VT, USA). The SNpc was defined by anatomical hallmarks. Rostrally, it began near the first TH^+^ cells at the end of the STN and extended caudally to the retrorubral field. Its boundaries were defined ventrolateral by the dorsal portion of the SN pars reticulata and anteromedial by the ventral tegmental area.^16,22^ Approximately seven sections spanning the entire SNpc were double-stained with TH and Nissl, with each section separated by 240 µm. The counting parameters included a grid size of 130 × 130 µm, a counting frame of 60 × 60 µm and a 1.5 µm guard zone. Sections were examined under a BX53 microscope with a 100×/1.25 numerical aperture objective (Olympus). The Gundersen coefficients of error for m = 1 were ≤0.10 for each section counted, except in cases of severe lesions.

### Statistics

Group analyses and correlation plots with linear regression were conducted in GraphPad Prism (version 9, GraphPad Software, San Diego, CA, USA). The Shapiro–Wilk normality test (α = 0.05) assessed data distribution. For normally distributed data among multiple groups, we used one-way ANOVA with *post hoc* Tukey’s test. Non-normally distributed data underwent a Kruskal–Wallis test followed by *post hoc* Dunn’s test. Pearson or Spearman correlation analyses were executed in Spyder 5.1.5 (Python 3.9), with pandas, numpy, seaborn, matplotlib, scipy and statsmodels libraries.

### Human subjects

For our translational experiments involving human data, we investigated seven subjects (one female, six males) with advanced PD and chronically implanted STN-DBS. The average disease duration was 11.0 ± 1.18 years, and mean age at the time of surgery was 67.43 ± 2.03 years. The average Unified Parkinson’s Disease Rating Scale (UPDRS) III score (Med-OFF) improved from 45.71 ± 5.10 points to 15.14 ± 2.03 (mean ± standard error of the mean) points by STN-DBS alone (Supplementary Table 1). The institutional review board of the University Hospital of Würzburg approved the study (registration no. 228/13 and 103/20). All involved subjects gave informed consent.

All patients received a single-photon computed tomography (SPECT) with ^123^I-*N*-ω-fluoropropyl-2β-carbomethoxy-3β-(4-iodophenyl)nortropane (FP-CIT) to measure the dopamine reuptake transporter (DAT) density prior to electrophysiology investigations (4.63 ± 3.89 months). SPECT data acquisition and analysis have been described previously.^23^ Individual striatal DAT binding measurements of patients were automated using the Brain Analysis Software (BRASS; Hermes Medical Solutions). Standardized 3D volume-of-interest (VOI) map measured semi-quantitative values of the specific radiotracer binding and ratios of striatum, caudate and putamen were calculated with the occipital cortex serving as the reference region [specific binding_striatum_ = (striatum—occipital reference)/occipital reference].

Electrophysiology recordings were performed in the Med-OFF condition with subjects at rest. LFPs were recorded with a single bipolar differential electrode configuration around the clinically defined cathode for each STN with a sampling rate of 422 Hz using the implanted IPG (Activa PC + S), 3389 leads, Medtronic PLC. High density EEG (hdEEG) signals were acquired using a 128-channel amplifier (BrainAmp, Brain Product) with a sampling rate of 1000 Hz. Both LFPs and EEGs were synchronized using an external TENS (Transcutaneous Electrical Nerve Stimulation) device by producing an artefact at 130 Hz, which was subsequently removed during preprocessing. Signal preprocessing and artefact rejection were performed using custom MATLAB scripts (v2023b, MathWorks) and the FieldTrip toolbox.^19^ All LFPs and EEGs were downsampled to 250 Hz prior to any preprocessing. LFP preprocessing was broadly as described previously.^24^ EEG preprocessing followed the steps in the EEGLAB pipeline.^25^ For EEG source analysis, co-registered [to the Montreal Neurological Institute (MNI) Colin27^26^ template] individual T1 MPRAGE MRIs using SPM12 Statistical Parametric Mapping (http://www.fil.ion.ucl.ac.uk/spm) were used to create a Finite Element Method (FEM) volume conduction model of the head. Inverse modelling was performed using a linear constrained minimum-variance (LCMV) beamformer, which estimates the activity of the brain for each source location (coordinates collected from the AAL3 atlas27) and finds a projection of the observed signal while minimizing interference from other signals for each source location.^28^ For the presented study, we only considered time series from the precentral gyrus (i.e. MCx) for both hemispheres. To generate comparable data, we analysed the human EEG and STN-LFP data using the Hanning window, consistent with the approach applied to the rodent data, rather than the method used in our earlier work.^29^

### Structural equation modelling

#### Model construction

We utilized the SEM toolbox for MATLAB (version 2020b, Mathworks, Natick, MA, USA) to perform structural equation modelling (SEM), a sophisticated analytical method for determining causal relationships among variables in a model-based approach.

##### Rodent data

In the first model, we examined the link between high β frequency features (MCx—high β power, high β burst amplitude; STN—high β power, high β burst amplitude), behaviour scores (cylinder test), striatal OD and TH^+^ SNpc dopamine neuron numbers across all three groups (EV, A53T-low, A53T-high). The second model explored the relationship between low β frequency features and the same output variables as the first model. For the third model, we devised a two-step procedure to establish a direct predictive model for each group separately and determine the incremental diagnostic value of output biomarkers for neurodegeneration. First, we normalized each parameter to its mean value before calculating cumulative sums (across the measured time points) for each group. Second, we constructed a composite score by minimizing errors for combinations within each group and assigning weights accordingly.^30^ We investigated the association between 22 estimated features from the MCx and STN (e.g. total β power, high β power, low β power, low β burst amplitude, burst rate, long burst probability) along with coherence (total β and high β) and behaviour scores, as well as the OD from the striatum, to predict the neurodegeneration composite score.

##### Human data

In the first model, we examined the relationship between high β-frequency features (specifically, high β power and burst amplitude from both the MCx and the STN) and clinical scores (UPDRS in the medication-OFF state) in a group of PD patients. The second model investigated the relationship between low β-frequency features, dopamine uptake in the caudate and putamen from both hemispheres as a mediator, and the same clinical outcome as in the first model.

#### Parameter estimation

We employed the maximum likelihood method for model fitting and used the root mean square error of approximation (RMSEA) index to adjust for longitudinal repeated measurements.^31^ Additionally, we assessed model fit using the invariant under a constant scaling (ICS) and ICS factor (ICSF) criteria, with lower values indicating better fit. The Akaike information criterion (AIC) was used to compare model quality, with smaller values indicating better fit. The obtained criterion comparing the models varied between 0.023 and 0.045 (which indicates a good fit for the models). The strength of associations between the variables in the models was quantified by standardized coefficients (S), ranging from 0 (no association) to 1 (very strong association). *P*-values <0.05 were considered significant. In addition to the AIC for the multiple models, we controlled the results; the adjusted Bonferroni correction severity of the adjustment was weakened with an increasing value of the average absolute correlation between two parameters in the model.^32^ The significance models described survived the adjusted Bonferroni correction with (*P* < 0.005). To visualize the prediction of the neurodegeneration composite score in the third model, a fitting approximation of the distribution was estimated for each rat. This was based on the probability distribution of each individual feature used in the prediction analyses.

## Results

### Dose-dependent dopaminergic neurodegeneration and motor impairment in the AAV-A53T-αSyn PD rat model

Taking advantage of a viral vector-mediated disease model, we applied two distinct virus titre concentrations to manipulate pathology severity and outcomes. Histological evaluation was conducted 8 weeks after AAV injection (Fig. 1 and Supplementary Fig. 1A and B). As anticipated, administration of a higher AAV1/2-A53T-αSyn titre (15 × 10^12^ gp/ml; A53T-high) induced more pronounced degeneration of dopaminergic (TH^+^) and total SNpc neurons (Nissl^+^ cells) compared to the lower titre (2.55 × 10^12^ gp/ml; A53T-low), with approximately 76% and 27%, respectively, for TH^+^ and 44% and 14% for Nissl^+^ cells, relative to the EV control group (Fig. 1B–D). Analysis of dopaminergic fibre density in the striatum revealed a corresponding decline of approximately 84% (A53T-high) and 48% (A53T-low), with respect to EV (Fig. 1E and Supplementary Fig. 1A). To evaluate the functional impact of varying levels of dopaminergic neurodegeneration in the AAV-A53T-αSyn PD rat model, we performed longitudinal motor function evaluations using the cylinder test. A53T-high and A53T-low PD rat models exhibited a higher preference for using the right forepaw, ipsilateral to the SNpc injection site, compared to EV controls, starting at Week 2 for the A53T high group and Week 4 for the A53T-low group (Fig. 1F). At the time point of 8 weeks after AAV injection, significantly greater motor impairment was evident in the A53T-high compared to the A53T-low PD rat model and EV (Fig. 1F). These data demonstrated that the severity of neurodegeneration depended on the dose of AAV1/2-A53T-αSyn viral vector in A53T PD rat models.

### Evolution of pathological β oscillatory activities in the AAV-A53T-αSyn PD rat model

**Figure 2:**
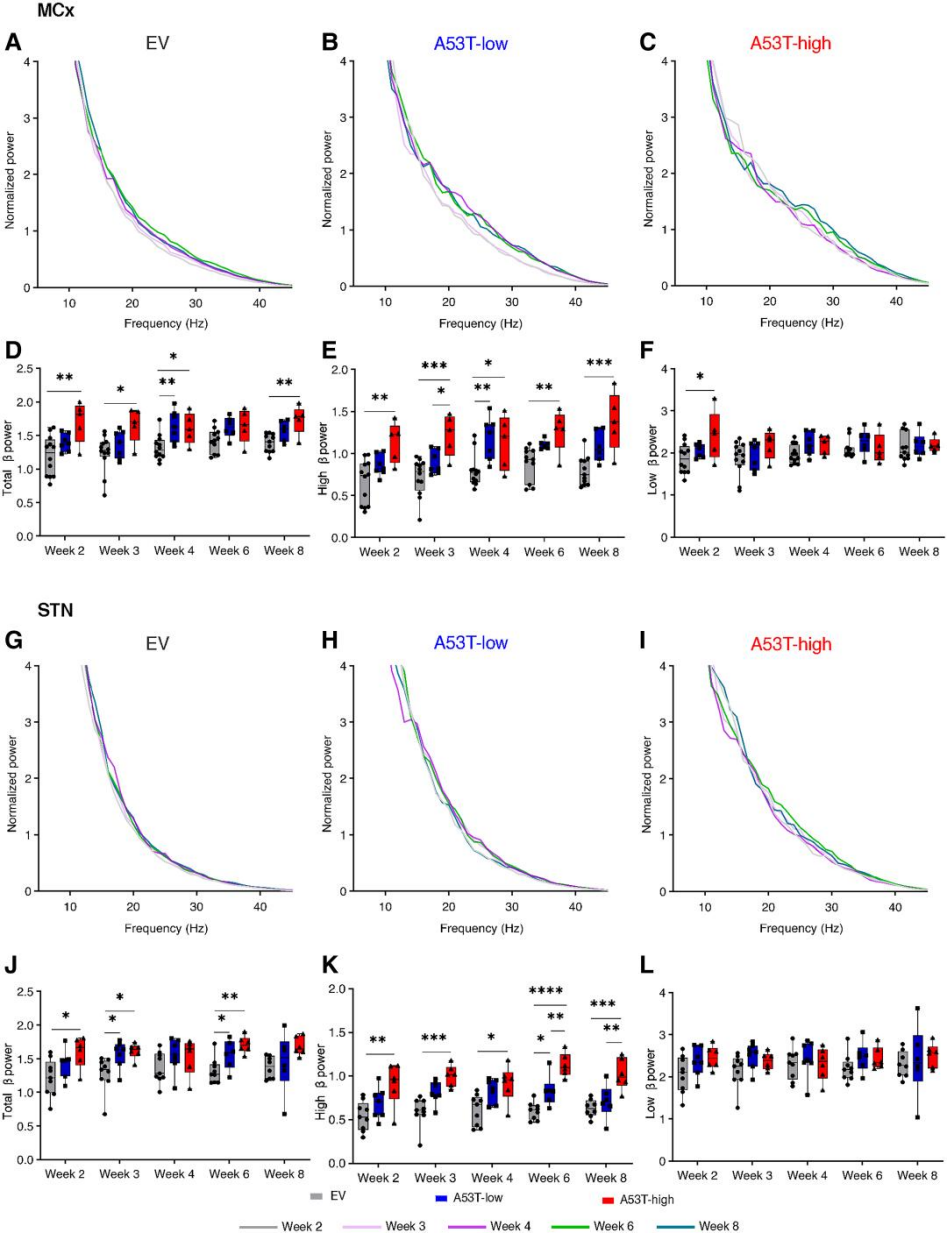
**Evolution of β power over 8** **weeks**. The first row displays group-averaged normalized power spectra within the frequency range of 5–45 Hz frequency range in the MCx of EV (**A**), A53T-low (**B**) and A53T-high rats (**C**). **D** represents the mean normalized MCx total β power (13–30 Hz) over 8 weeks [Week 2: *F*(2,21) = 6.811, *P* = 0.0052; Week 3: *F*(2,21) = 4.932, *P* = 0.0175; Week 4: *F*(2,21) = 6.604, *P* = 0.0060; Week 6: *F*(2,19) = 3.296, *P* = 0.0590; Week 8: *F*(2,19) = 8.720, *P* = 0.0021]. **E** represents the mean normalized MCx high β power (21–30 Hz) over 8 weeks [Week 2: *F*(2,21) = 8.052, *P* = 0.0025; Week 3: *F*(2,21) = 10.30, *P* = 0.0008; Week 4: *X^2^* = 10.77, *P* = 0.0046; Week 6: *F*(2,19) = 8.340, *P* = 0.0025; Week 8: *F*(2,19) = 12.10, *P* = 0.0004]. **F** represents the mean normalized MCx low β power (13–20 Hz) over 8 weeks [Week 2: *F*(2,21) = 3.966, *P* = 0.0346; Week 3: *F*(2,21) = 1.363, *P* = 0.2777; Week 4: *F*(2,21) = 2.955, *P* = 0.0740; Week 6: *X^2^* = 1.979, *P* = 0.3877; Week 8: *F*(2,19) = 0.08636, *P* = 0.9176]. *n*_rats_ in **D**–**F**, Weeks 2–4: *n* = 12 for EV, *n* = 7 for A53T-low, *n* = 5 for A53T-high; Weeks 6 and 8: *n* = 11 for EV, *n* = 6 for A53T-low, *n* = 5 for A53T-high. (**G**–**I**) Group-averaged normalized power spectra in the 5–45 Hz frequency range in the STN of EV (**G**), A53T-low (**H**) and A53T-high rats (**I**). **G** shows box plots of mean normalized STN total β power (13–30 Hz) over 8 weeks [Week 2: *F*(2,19) = 4.488, *P* = 0.0253; Week 3: *X^2^* = 9.415, *P* = 0.0047; Week 4: *F*(2,19) = 1.291, *P* = 0.2980; Week 6: *F*(2,18) = 8.903, *P* = 0.0021; Week 8: *F*(2,17) = 2.325, *P* = 0.1280]. **H** represents the mean normalized STN high β power (21–30 Hz) over 8 weeks [Week 2: *F*(2,19) = 6.287, *P* = 0.0080; Week 3: *X^2^* = 14.27, *P* < 0.0001; Week 4 *F*(2,19) = 6.153, *P* = 0.0087; Week 6: *F*(2,18) = 27.72, *P* < 0.0001; Week 8: *F*(2,17) = 11.05, *P* = 0.0008]. **I** represents the mean normalized STN low β power (13–20 Hz) over 8 weeks [Week 2: *F*(2,19) = 2.419, *P* = 0.1159; Week 3: *F*(2,19) = 2.542, *P* = 0.1052; Week 4: *F*(2,19) = 0.2510, *P* = 0.7806; Week 6: *F*(2,18) = 1.586, *P* = 0.2321; Week 8: *F*(2,17) = 0.2911, *P* = 0.7511]. *n*_rats_ in **J**–**L**, Weeks 2–4: *n* = 9 for EV, *n* = 7 for A53T-low, *n* = 6 for A53T-high; Week 6: *n* = 9 for EV, *n* = 6 for A53T-low, *n* = 6 for A53T-high; Week 8: *n* = 8 for EV, *n* = 6 for A53T-low, *n* = 6 for A53T-high. Box plot data are shown as: whiskers + min to max. *P < 0.05, **P < 0.01, ***P < 0.001 and ****P < 0.0001 for the *post hoc* comparisons of one-way ANOVA or Kruskal–Wallis test. EV = empty vector; MCx = motor cortex; STN = subthalamic nucleus.

To assess whether the A53T PD rat models developed pathological LFP signals over time, we conducted simultaneous recordings from the MCx and STN over an 8-week period. Three rats were excluded from STN signal analysis because the electrode localization was off target, as verified by Nissl staining (Supplementary Fig. 1C). One rat was excluded from MCx analysis due to artefacts. To characterize the β evolution, power spectra were first analysed, followed by a mean β power calculation within the total, high and low β range (Fig. 2). The A53T-high PD rat model displayed a significant increase in both MCx and STN total β power from Week 2 onwards (Fig. 2D and J). In contrast, the A53T-low PD rat model only exhibited a transient elevation of total β power (13–30 Hz), which did not reach significance until Week 3 (STN), Week 4 (MCx) and Week 6 (STN), suggesting a less consistent pathology progression (Fig. 2D and J). Interestingly, the elevation of β power was primarily attributed to an increase in the high β range (21–30 Hz), specifically in the A53T-high PD rat model (Fig. 2E and K); meanwhile, with the exception of Week 2 for the A53T-high group (MCx), the low β power (13–20 Hz) did not show any significant differences when comparing all three groups of rats (Fig. 2F and L).

**Figure 3:**
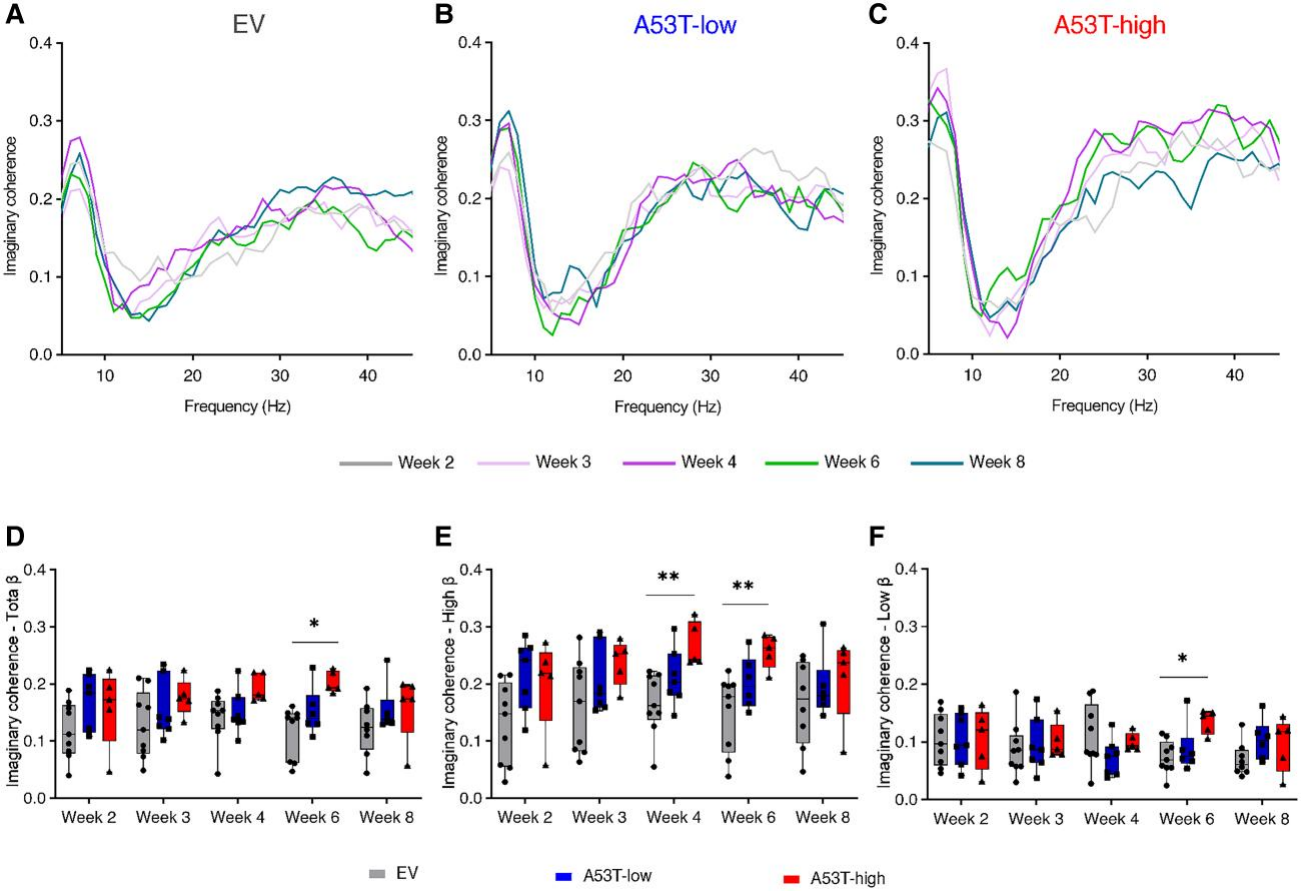
**MCx-STN β imaginary coherence over 8 weeks**. (**A**–**C**) MCx-STN coherence spectra of EV (**A**), A53T-low (**B**) and A53T-high rats (**C**) over 8 weeks. Box plots represent the mean coherence power in total β (13–30 Hz, **D**), high β (21–30 Hz, **E**) and low β range (13–20 Hz, **F**) [Week 2—total: *F*(2,18) = 1.748, *P* = 0.2024; high: *F*(2,18) = 3.163, *P* = 0.0665; low: *F*(2,18) = 0.0044, *P* = 0.9956; Week 3—total: *F*(2,18) = 1.489, *P* = 0.2521; high: *X^2^* = 3.306, *P* = 0.1969; low: *F*(2,18) = 0.1448, *P* = 0.8662; Week 4—total: *F*(2,18) = 3.124, *P* = 0.0684; high: *F*(2,18) = 6.755, *P* = 0.0065; low: *F*(2,18) = 1.662, *P* = 0.2176; Week 6—total: *X^2^* = 8.69, *P* = 0.0071; high: *F*(2,17) = 6.371, *P* = 0.0086, low: *X^2^* = 7.302, *P* = 0.0189; Week 8—total: *X^2^* = 3.28, *P* = 0.2000; high: *X^2^* = 1.641, *P* = 0.4602; low: *F*(2,16) = 1.710, *P* = 0.2123]. *n*_rats_ in **D**–**F**, Weeks 2–4: *n* = 9 for EV, *n* = 7 for A53T-low, *n* = 5 for A53T-high; Week 6: *n* = 9 for EV, *n* = 6 for A53T-low, *n* = 5 for A53T-high; Week 8: *n* = 8 for EV, *n* = 6 for A53T-low, *n* = 5 for A53T-high. Box plot data are presented as: whiskers + min to max. *P < 0.05, **P < 0.01, ***P < 0.001 and ****P < 0.0001 for the *post hoc* comparisons after one-way ANOVA or Kruskal–Wallis test. EV = empty vector; MCx = motor cortex; STN = subthalamic nucleus.

To further characterize the β synchronization between the MCx and STN, the imaginary coherence spectra and the mean coherence values within the total, high and low β frequency ranges were calculated (Fig. 3). A significant increase in total β coherence was observed in the A53T-high PD rat model group when compared to EV controls at Week 6 after AAV injection (Fig. 3D). Analysis of high β coherence displayed significant elevation in the A53T-high PD rat model at Weeks 4 and 6 (Fig. 3E). Significant coherence was also found for low β comparing EV with A53T-high rats at Week 6 (Fig. 3F). The A53T-low PD rat model did not develop any significant elevation of coherence.

**Figure 4:**
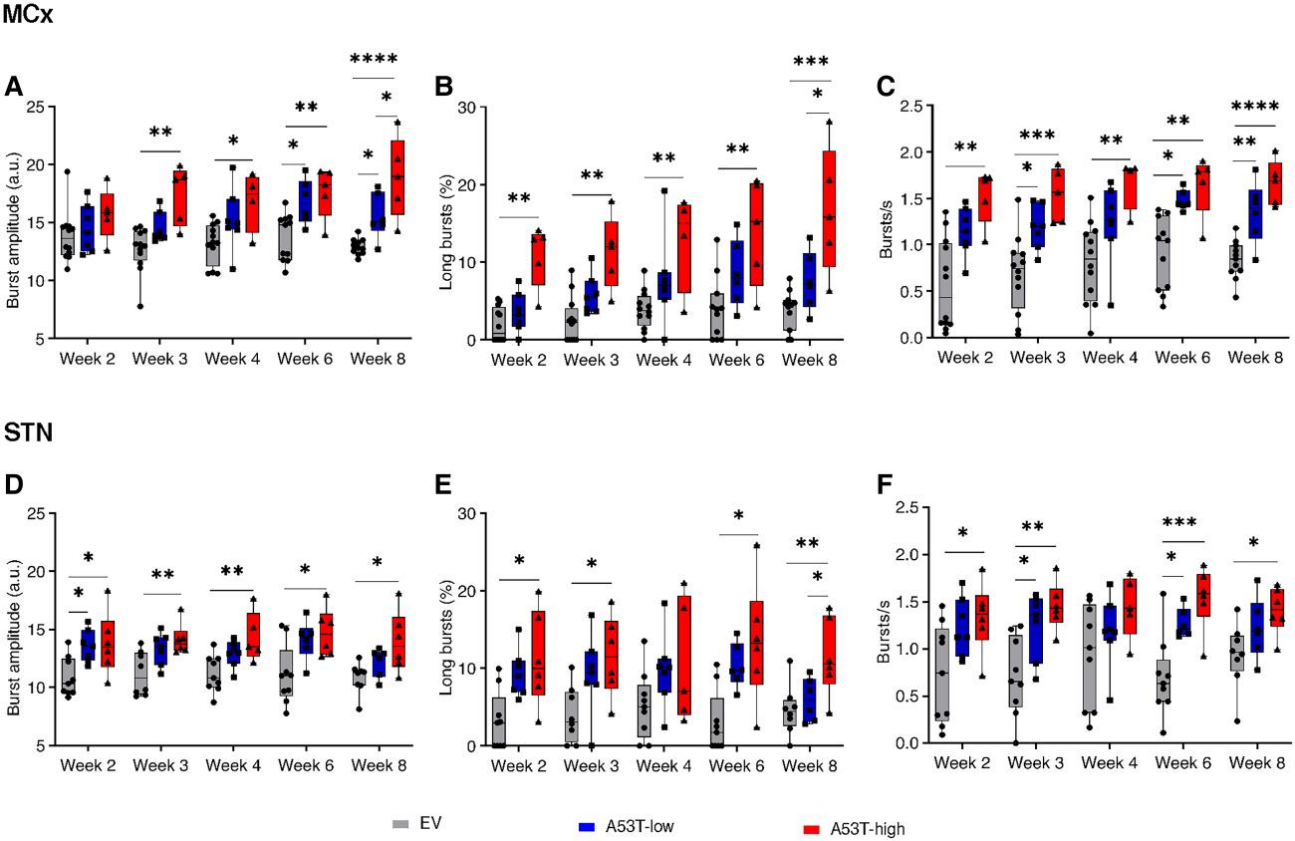
**High β (21–30 Hz) burst dynamics over 8 weeks**. High β-burst amplitude (**A**), long-burst probability (**B**) and burst rate (**C**) in the MCx of EV (grey), A53T-low (blue) and A53T-high rats (red) over 8 weeks (Week 2—burst amplitude: *X^2^* = 3.574, *P* = 0.1675; burst rate: *X^2^* = 12.39, *P* = 0.0020; long bursts: *X^2^* = 10.50, *P* = 0.0053; Week 3—burst amplitude: *X^2^* = 10.25, *P* = 0.0060; burst rate: *F*(2,21) = 11.64, *P* = 0.0004; long bursts: *X^2^* = 12.07, *P* = 0.0024; Week 4—burst amplitude: *F*(2,20) = 4.900, *P* = 0.0185; burst rate: *X^2^* = 10.03, *P* = 0.0066; long bursts: *F*(2,20) = 6.428, *P* = 0.0070; Week 6—burst amplitude: *F*(2,19) = 7.962, *P* = 0.0031; burst rate: *X^2^* = 13.48, *P* < 0.0001; long bursts: *F*(2,19) = 7.269, *P* = 0.0045; Week 8—burst amplitude: *F*(2,19) = 15.98, *P* < 0.0001; burst rate: *F*(2,19) = 20.78, *P* < 0.0001; long bursts: *F*(2,19) = 13.16, *P* = 0.0003). *n*_rats_ in **A**–**C**, Weeks 2 and 3: *n* = 12 for EV, *n* = 7 for A53T-low, *n* = 5 for A53T-high; Week 4: *n* = 12 for EV, *n* = 7 for A53T-low, *n* = 4 for A53T-high; Weeks 6 and 9: *n* = 11 for EV, *n* = 6 for A53T-low, *n* = 5 for A53T-high. (**D**–**F**) High β-burst amplitude (**D**), long burst probability (**E**) and burst rate (**F**) in the STN of EV (grey), A53T-low (blue) and A53T-high rats (red) over 8 weeks [Week 2—burst amplitude: *F*(2,19) = 5.729, *P* = 0.0113; burst rate: *F*(2,19) = 4.447, *P* = 0.0261; long bursts: *X^2^* = 9.252, *P* = 0.0054; Week 3—burst amplitude: *F*(2,18) = 6.569, *P* = 0.0072; burst rate: *F*(2,19) = 8.838, *P* = 0.0019; long bursts: *F*(2,18) = 5.086, *P* = 0.0177; Week 4—burst amplitude: *F*(2,18) = 6.433, *P* = 0.0078; burst rate: *F*(2,18) = 2.021 *P* = 0.1615; long bursts: *F*(2,18) = 2.165, *P* = 0.1437; Week 6—burst amplitude: *F*(2,18) = 5.808, *P* = 0.0113; burst rate: *F*(2,18) = 12.12, *P* = 0.0005; long bursts: *X^2^* = 9.768, *P* = 0.0035; Week 8—burst amplitude: *F*(2,17) = 5.083, *P* = 0.0186; burst rate: *F*(2,17) = 4.070, *P* = 0.0360; long bursts: *F*(2,17) = 6.222, *P* = 0.0094]. *n*_rats_ in **D**–**F**, Weeks 2 and 3: *n* = 9 for EV, *n* = 7 for A53T-low, *n* = 6 for A53T-high (in **D** and **E** Week 3 EV *n* = 8); Week 4: *n* = 9 for EV, *n* = 7 for A53T-low, *n* = 5 for A53T-high; Week 6: *n* = 9 for EV, *n* = 6 for A53T-low, *n* = 6; Week 8: *n* = 8 for EV, *n* = 6 for A53T-low, *n* = 6. Box plot data are shown as: whiskers + min to max. *P < 0.05, **P < 0.01, ***P < 0.001 and ****P < 0.0001 for *post hoc* comparisons after one-way ANOVA or Kruskal–Wallis test. EV = empty vector; MCx = motor cortex; STN = subthalamic nucleus.

Given the important role of pathological β bursting in LFP recordings from human PD patients in the context of pre-movement bursts and motor control, we next focused on the analysis of high β burst amplitude, rate and proportion of long bursts in the AAV-A53T-αSyn PD rat model and controls (Fig. 4). In A53T-high rats, there was a notable increase in burst amplitude in MCx compared to EV rats, becoming significant from Week 3 onwards (Fig. 4A). With a delay, the A53T-low PD rat model demonstrated an elevation of burst amplitude from Week 6 onward (Fig. 4A). In contrast, a significant increase in burst rate and proportion of long bursts was observed in the A53T-high PD rat model as early as 2 weeks after AAV administration (Fig. 4B and C). Once again, A53T-low rats demonstrated delayed emergence of β-burst alteration, displaying an irregular elevation at Weeks 3 and 8 after AAV delivery (Fig. 4C). Comparable results were observed when analysing high β bursts in the STN (Fig. 4D–F). Specifically, an early increase in burst amplitude was detected at Week 2 in both PD groups (Fig. 4D). This elevation persisted consistently over 8 weeks in the A53T-high group, whereas it was transient in the A53T-low group, being observed only at Week 2.

### High β activities in the STN but not MCx correlated with motor impairment in the AAV-A53T-αSyn PD rat model

**Figure 5:**
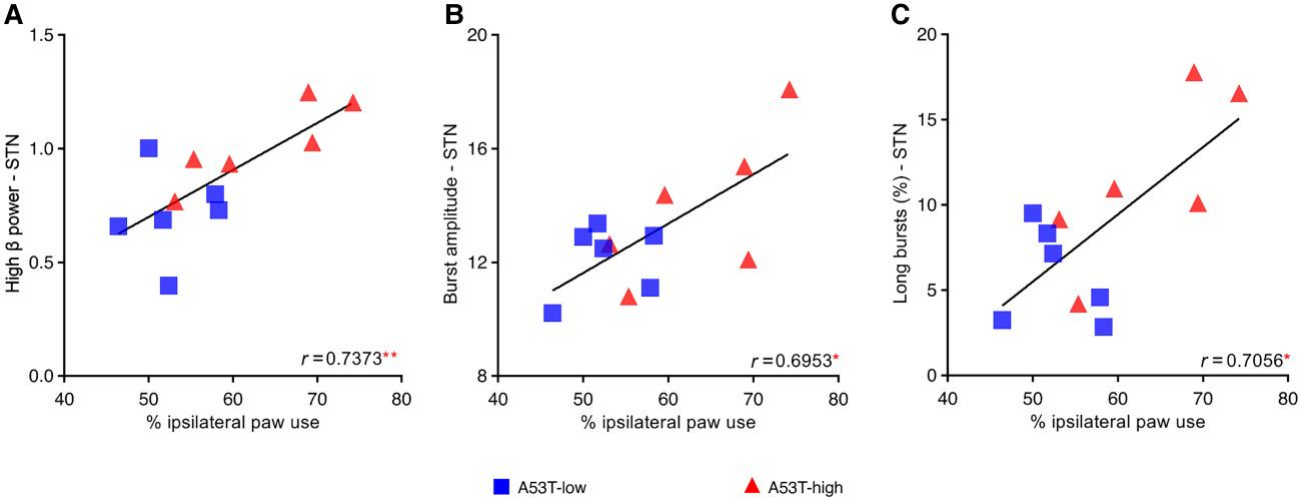
**Correlation analyses of high β (21–30 Hz) power with motor deficit in the AAV-A53T-αSyn Parkinson’s disease rat model**. Motor deficit (cylinder test) is related to high β-power (**A**), burst amplitude (**B**) and long burst probability (**C**) of STN (blue squares: A53T-low *n* = 6, red triangles: A53T-high *n* = 6). All correlation (*r*) values and *P*-values are summarized in Table 1. *P < 0.05, **P < 0.01, ***P < 0.001 and ****P < 0.0001 for correlation analyses. STN = subthalamic nucleus.

We next aimed to elucidate the interplay between pathologically enhanced β power and the severity of motor deficits. Correlation analyses at Week 8 uncovered a strong correlation between high β power in the STN and motor impairment (Fig. 5A and Table 1; *r* = 0.7373), while the correlation between high β power in the MCx and motor impairment was not significant (Table 1). Of note, highly significant correlations of motor deficits with high β burst parameters at Week 8 were exclusively found within the STN, in particular for burst amplitude (Fig. 5B and Table 1; *r* = 0.6953) and the percentage of long bursts (Fig. 5C and Table 1; *r* = 0.7056), but not for burst rate (Table 1). This indicated a strong association between various burst parameters in the high β band recorded from the STN with motor impairment in the AAV-A53T-αSyn PD rat model. In contrast, low β power and burst parameters in both the MCx and STN showed no associations to motor deficits (Supplementary Table 2).

**Table 1:** Relationship between high β parameters, motor dysfunction and histological outcome measures

Regions	LFP parameters	*r*-value	*P*-value
**Motor deficit correlations with high β parameters at Week 8**			
MCx	Power	0.3882	0.2381
	Burst amplitude	0.4528	0.1619
	Long bursts, %	0.4360	0.1800
	Burst rate	0.1477	0.6648
STN	Power	0.7373	0.0062
	Burst amplitude	0.6953	0.0121
	Long bursts, %	0.7056	0.0104
	Burst rate	0.2734	0.3899
**Number of TH^+^ SNpc neurons correlations with high β parameters**			
MCx	Power	−0.4525	0.1623
	Burst amplitude	−0.3782	0.2515
	Long bursts, %	−0.4922	0.1241
	Burst rate	−0.4175	0.2014
STN	Power	−0.7132	0.0092
	Burst amplitude	−0.4336	0.1590
	Long bursts, %	−0.6508	0.0219
	Burst rate	−0.1949	0.5439
**Striatal dopaminergic fibre density correlations with high β parameters**			
MCx	Power	−0.4661	0.1484
	Burst amplitude	−0.3575	0.2805
	Long bursts, %	−0.5321	0.0920
	Burst rate	−0.4807	0.1344
STN	Power	−0.5946	0.0414
	Burst amplitude	−0.3905	0.2095
	Long bursts, %	−0.6371	0.0259
	Burst rate	−0.2717	0.3929

LFP = local field potential; MCx = motor cortex; SNpc = substantia nigra pars compacta; STN = subthalamic nucleus; TH = tyrosine hydroxylase.

### High STN β activity, but not MCx, correlated with dopaminergic degeneration in the PD rat model

**Figure 6:**
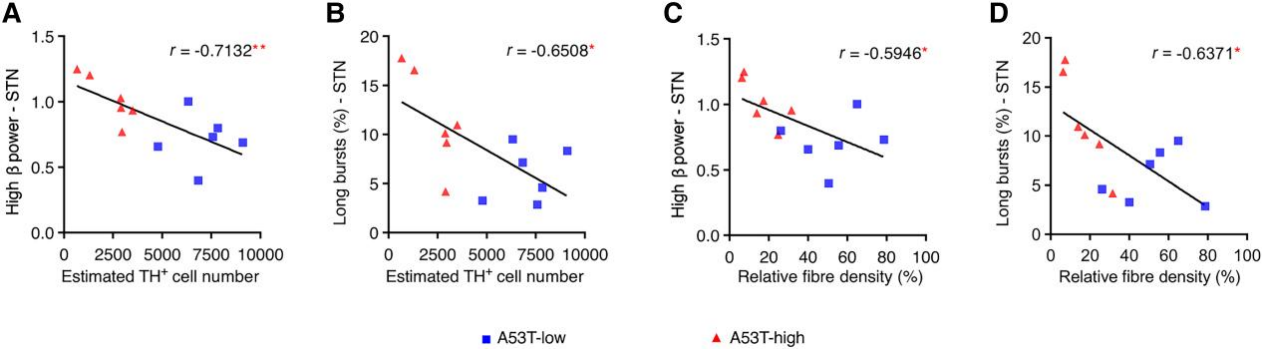
**Correlation analyses of high β (21–30 Hz) parameters with nigrostriatal denervations in the AAV-A53T-αSyn Parkinson’s disease rat model**. TH^+^ neuron numbers in SNpc are related to STN high β power (**A**) and the long burst probability (**B**) but not burst amplitude and burst rate (Table 1). Dopaminergic fibre density in striatum is related to STN high β power (**C**) and the long burst probability (**D**) but not burst amplitude and burst rate (A53T-low *n* = 6, A53T-high *n* = 6; Table 1). All *r*- and *P*-values are summarized in Table 1. *P < 0.05, **P < 0.01, ***P < 0.001 and ****P < 0.0001 for correlation analyses. SNpc = substantia nigra pars compacta; STN = subthalamic nucleus; TH = tyrosine hydroxylase.

When examining the correlation between the quantity of TH^+^ SNpc neurons and either MCx or STN high power, recorded at Week 8, we observed a significantly negative correlation, specifically with β parameters in STN—such as high β power (Fig. 6A and Table 1; *r* = −0.7132) and long burst probability (Fig. 6B and Table 1; *r* = −0.6508). The density of striatal dopaminergic fibres exhibited a negative correlation with both STN high β power (Fig. 6C and Table 1; *r* = −0.5946) and long burst probability (Fig. 6D and Table 1; *r* = −0.6371). Conversely, the analysis of high β parameters in the MCx concerning either TH^+^ SNpc neuron number or striatal dopaminergic fibre density did not reveal any significant correlations (Table 1). Furthermore, low β power and low β burst parameters in both regions did not show significant correlations with TH^+^ neuron number in SNpc and dopaminergic OD in the striatum (Supplementary Tables 3 and 4).

### Multivariate analyses of β parameters predicted motor decline and dopaminergic loss in the PD model

**Figure 7:**
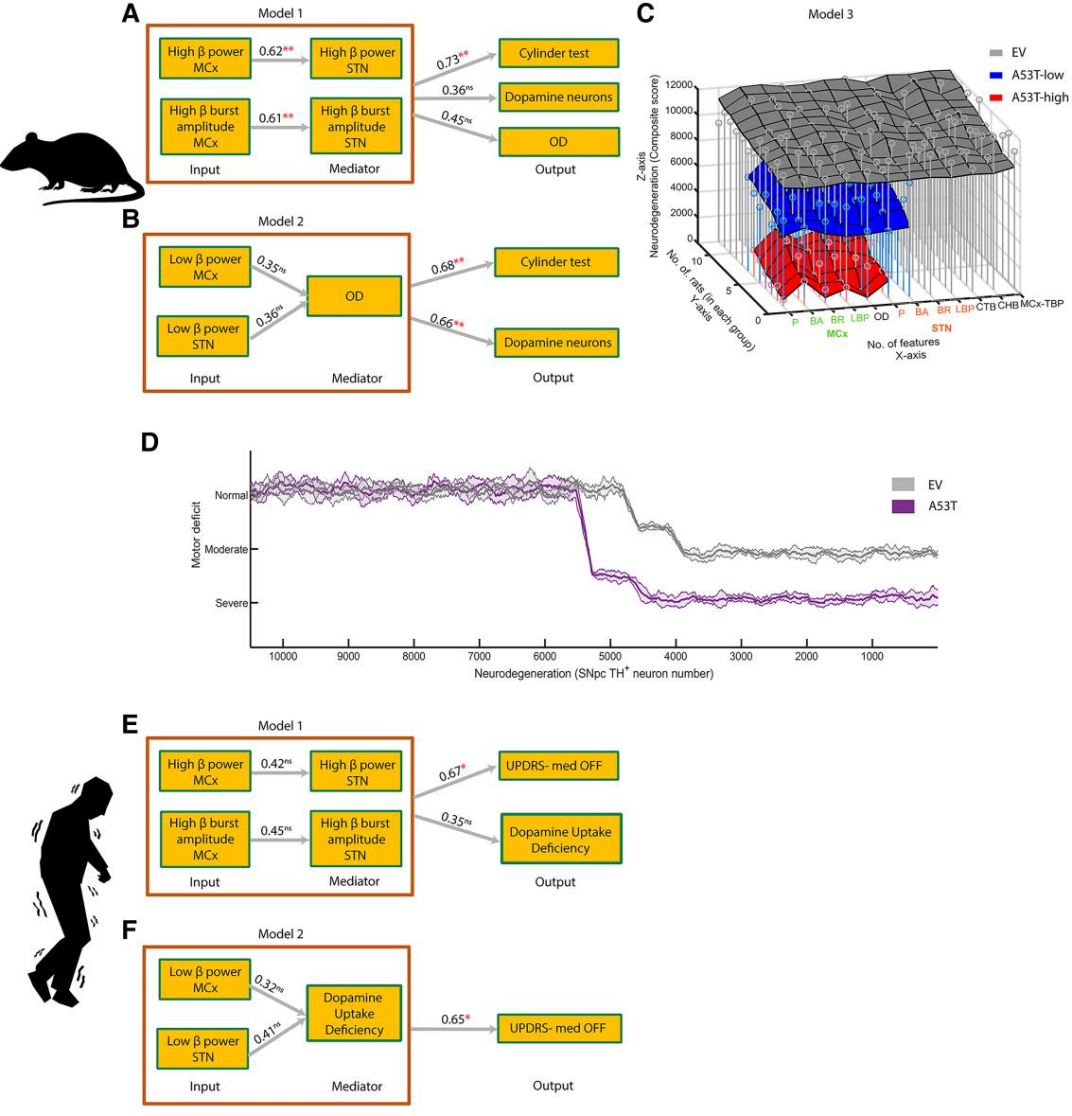
**Multivariate analysis and neurodegeneration composite score.** In the first model (**A**), relationships between changes in the high β power and burst amplitude from the MCx as the input with the high β power and burst amplitude from the STN as mediators predicted motor performance from the cylinder test. In contrast, the number of dopaminergic SNpc neurons and the TH^+^ striatal OD were not significantly predicted. In the second model (**B**), low β power from the MCx and the STN acted as the input and the OD acted as a mediator. This combination significantly predicted motor performance from the cylinder test and the dopamine neuron numbers in SNpc. (**C**) The three groups, namely EV (grey), A53T-low (blue) and A53T-high (red), are shown. The stem plot in each colour represents the actual values for each group, and the sheets show the predicted values for each group. The features used in the predictions for each rat are shown on the *x*-axis, based on the best predictor first: low β power (P), burst amplitude (BA), burst rate (BR), long burst probability (LBP) in the MCx, followed by the STN features: P, BA, BR, LBP, coherence total β (CTB), coherence high β (CHB), MCx total β power (MCx-TBP) and striatal OD. MCx signal parameters are labelled in green and STN in orange. The *y*-axis depicts the number of rats in each group, and the *z*-axis depicts the neurodegeneration (composite score). (**D**) The survival probability curves, which relate behaviour and neurodegeneration for the two groups separately (namely EV, A53T, considering all the longitudinally measured time points), are shown. *n*_rats_: high/low β power MCx *n* = 24; high/low β power STN *n* = 22; high beta burst amplitude MCx *n* = 24; high β burst amplitude STN *n* = 22; cylinder test score, dopamine neurons and OD values *n* = 25. (**E**) In the first (human) model, changes in the relationship between high β power and the high β burst amplitude from the MCx as the input, along with high β power and high β burst amplitude from the STN as mediators, were robustly predicted clinical motor scores (UPDRS) in the Med-OFF condition but failed to predict dopamine uptake. In the second (human) model, (**F**) low β power from the MCx and the STN with striatal dopamine uptake as the mediator shows a robust association with clinical motor scores (UPDRS) in the Med-OFF condition. *n*_human_ = 7. The behaviour values were *z*-normalized per group and decoded to normal, moderate and severe deficits in behaviour. *P* < 0.05 followed by Bonferroni correction (**P < 0.005) for multivariate analysis. EV = empty vector; MCx = motor cortex; OD = optical density; UPDRS = Unified Parkinson’s Disease Rating Scale; SNpc = substantia nigra pars compacta; STN = subthalamic nucleus; TH = tyrosine hydroxylase.

We next implemented a multivariate analysis using SEM and generated three models (Fig. 7A–C). The fit indices obtained for all three models implied a good fit of the constructed models to the observed data, providing robust causal relationships among the variables. In the first model (Fig. 7A), the incorporation of high β power values from the MCx, alongside high β burst amplitude from the MCx (input), with identical parameters from STN as a mediator, emerged as robust predictors for the behavioural scores (S = 0.73; *P* < 0.005). However, the first model failed to predict both the number of dopaminergic neurons in the SNpc (S = 0.36; *P* > 0.05) and the dopaminergic fibre density (OD) in the striatum (S = 0.45; *P* > 0.05; Fig. 7A). Interestingly, from the first model we found that high β power in the MCx predicted the high β power in the STN (S = 0.62; *P* < 0.005). In addition, high β burst amplitudes of the MCx were predictive for the high β burst amplitudes of the STN (S = 0.61; *P* < 0.005; Fig. 7A).

In the second model (Fig. 7B), low β power from both the MCx and STN served as input variables. When coupled with striatal TH^+^ OD as a mediator, they exhibited a robust association with both motor performance (S = 0.68; *P* < 0.005) and the number of dopaminergic SNpc neurons (S = 0.66; *P* < 0.005; Fig. 7B). In the third model (Fig. 7C), the combination of four features from the MCx namely low β power (S = 0.34), burst amplitude (S = 0.20), burst rate (S = 0.15) and long burst probability (S = 0.13), in addition to TH^+^ OD (S = 0.10) and low β power (S = 0.08) from the STN demonstrated strong predictive power for neurodegeneration (measured by a composite score) in the A53T-high group. Conversely, additional features were required for the A53T-low group to predict neurodegeneration, specifically the STN low β burst amplitude (S = 0.52) and burst rate (S = 0.48). For the EV group, four additional features were needed, including the STN low burst probability (S = 0.37), total β (S = 0.29) and high β coherence (S = 0.23), along with the MCx total β power (S = 0.11). These findings highlight the distinct predictive roles of high versus low β activity: while high β activity predicts motor impairment, low β activity—in concert with striatal dopaminergic denervation—predicts both motor impairment and SNpc dopaminergic neuron loss. Upon integrating our neurodegeneration data with behavioural assessments, we endeavored to construct a final model aimed at predicting behavioural deficits in relation to neurodegeneration (Fig. 7D). We observed a sharp decline in motor function in the A53T-high PD rat model when dopaminergic neurodegeneration reached approximately 50% (Fig. 7D). Given the nearly linear progression of neurodegeneration over time in the A53T PD rat model, we postulated that compensatory mechanisms operated to forestall motor decline up to a certain threshold of neurodegeneration. Intriguingly, EV control rats were predicted to exhibit motor deficits only at a higher level of neurodegeneration compared to the A53T PD rat model (Fig. 7D). These findings suggested that non-PD-affected EV rats possessed greater compensatory capabilities compared to A53T PD diseased rats.

### Multivariate analyses using β parameters translationally predicted motor impairment in human PD patients

To evaluate the translational relevance of our findings from the A53T PD rat model, we applied our newly developed multivariate analyses using β parameters to a dataset of human PD patients. The fit indices obtained for two human models implied a good fit of the constructed models to the observed data, providing robust causal relationships among the variables (Fig. 7E and F and Supplementary Fig. 2). In the first model for human PD patients, the incorporation of high β power values from MCx alongside high β burst amplitude from MCx (input) with identical parameters from STN as a mediator, emerged as robust predictors for the clinical UPDRS III motor Med-OFF score (standardized coefficient S = 0.67; *P* < 0.01; Fig. 7E). In the second model, low β power from both the MCx and STN served as input variables. When coupled with striatal dopamine uptake from the caudate and putamen as a mediator, they exhibited a robust association with UPDRS III motor Med-OFF (S = 0.65; *P* < 0.01; Fig. 7F).

## Discussion

Animal disease models offer the advantage of enabling standardized investigations, allowing studies to be conducted on the occurrence of various pathology-relevant changes and their interconnections. Leveraging this advantage, we performed a longitudinal and multidimensional assessment of histological, behavioural and electrophysiological pathological patterns in the AAV-A53T-αSyn PD rat model.

We utilized the benefit of a viral vector-mediated disease model and employed two distinct titer concentrations to modulate pathology severity and outcomes. In terms of neurodegeneration, we found a dose-dependent reduction in dopaminergic neurons within the SNpc in the PD models at Week 8 after AAV injection. Specifically, there was a 27% reduction in cell counts in the SNpc of the low-titre PD model and a more pronounced 76% reduction in the high-titre PD model, relative to the cell counts observed in the SNpc of the control group receiving EV. Moreover, we noticed a dose-dependent impact on motor impairments using the cylinder test in the AAV-A53T-αSyn PD rat model. This was evident by a significant increase in asymmetrical forepaw use in rats administered a high dose of A53T-αSyn-encoding AAV, starting as early as Week 2 after injection, compared to EV rats. Conversely, within the low-dose PD model, a significant decline in motor function became apparent after a delay, the earliest showing at the 4-week time point. A dose-dependent relationship in terms of the induction and expression of PD-like symptoms and dopaminergic neurodegeneration can also be observed in neurotoxic PD models. For instance, injection with increasing concentrations (ranging from 4–16 µg) of 6-hydroxydopamine (6-OHDA) into the medial forebrain bundle (MFB) of rats has been used to establish a graded model encompassing distinct clinical stages of PD. Notably, the injection of escalating 6-OHDA concentrations resulted in progressively more pronounced cell loss and correlating behavioural alterations.^33^

We conducted an electrophysiological analysis of LFPs in awake rats during the resting state. This approach offers a distinct advantage over brain recordings carried out under analgo-sedation (e.g. with isoflurane or urethane), which have been shown to influence the power of low-frequency oscillations.^34,35^ We observed an early elevation of β power within the MCx and STN in the high-titre AAV-A53T-αSyn PD rat model from as early as Week 2 and within low-titre rats at Week 4, thereby highlighting that pathologically enhanced β oscillations manifest at an early stage in this PD model. Interestingly, this increase was predominantly attributed to an elevation in the 21–30 Hz range, termed the high β frequency range. This aligns with observations from the reserpine model of PD.^36^ In the 6-OHDA PD rat model, however, both high and low β power exhibited elevation in the STN.^36^ Of note, human studies have suggested that β activity can be categorized into two distinct frequency components, each possibly serving different functions.^37,38^ Low β activity (13–20 Hz) responds to dopaminergic medication and DBS, and correlates with PD severity of motor symptoms such as rigidity and bradykinesia—it is thus regarded as a marker of the Parkinsonian network state.^39,40^ Conversely, the role of high β activity remains subject to ongoing discussion. On one hand, it has been linked to freezing of gait,^41^ while on the other, it has been associated with long-distance physiological coupling between distinct brain regions. This notion is supported by the frequent observation of coupling between STN high β and cortical high-frequency oscillations, which are considered physiologic.^42^ In our AAV-A53T-αSyn PD rat model, only β oscillations in the STN correlated with motor impairment. However, this correlation was specific to forelimb asymmetry, while freezing of gait was not specifically analysed. Moreover, we observed an increase in total, high and low β imaginary coherence between the STN and MCx as the model pathology progressed and severity increased, with the most notable rise occurring in high β coherence. Given that increased β coherence between the STN and sensorimotor cortex has been reported in PD patients,^43^ our findings provide additional insights into the role of high and low β oscillations in PD pathophysiology.

Recent studies have linked increased β power in PD to β bursts, thereby acknowledging the fast dynamics of oscillatory activity.^44,45^ STN recordings from PD patients without dopaminergic medication revealed manifestation of β bursts during repetitive movements and their connection with reduced movement velocity.^45^ Increased occurrence of long β bursts has been associated with the OFF state in PD patients and observed to increase progressively over time in the 6-OHDA PD model.^3,9^ These findings established a connection between β bursts during movement and bradykinesia. Based on this knowledge, we assessed the occurrence of β bursts in our PD model and found a significant correlation of long β burst probability with motor impairment, predominantly in the STN. This observation further emphasized the relevance of STN β bursts, particularly long bursts, as a valuable pathophysiological marker in PD.

Taking advantage of the extensive and longitudinal dataset obtained from our AAV-A53T-αSyn PD rat model, we employed SEM to identify whether our LFP data could be used to predict motor impairment and loss of dopaminergic terminals in the striatum or neurons in the SNpc. Intriguingly, both high β power and bursts recorded from the MCx and STN exhibited predictive capabilities for motor deficits in the PD rat model, but did not demonstrate predictive ability for dopaminergic end points. This observation underlines the significance of high β power in PD symptomatology. An additional, complimentary model from our study suggested the pivotal role of low β power in both the MCx and STN (in conjunction with striatal dopaminergic terminal loss) as potential predictive factors for both motor impairment and, notably, dopaminergic neuron loss in the SNpc of the AAV-A53T-αSyn PD rat model. Given that the correlation between pathological β power and SNpc dopaminergic neuron count has been demonstrated primarily in PD toxin models, but not in human PD,^3^ and considering conflicting outcomes of striatal dopamine transporter imaging in relation to SN neuron numbers in PD patients,^46,47^ our study suggests a translationally relevant method for predicting SNpc neuron loss in PD by combining measurements of low β oscillations with assessments of striatal dopaminergic levels. Given the nearly linear progression of neurodegeneration in the A53T-high PD rat model, we hypothesize that compensatory mechanisms delay motor decline until a critical neurodegeneration threshold is reached. In contrast, EV control rats exhibited motor deficits only at more advanced levels of neurodegeneration compared to the AAV-A53T-αSyn PD rat model, suggesting that EV rats possess a greater compensatory capacity in the absence of PD-related pathology.

In conclusion, we combined longitudinal assessments of LFPs from the STN and MCx in awake A53T PD model rats with behavioural analyses and neurodegenerative end points. Our results demonstrate a close trilateral interrelation between the evolution of pathological LFP features, motor symptom expression and the severity of dopaminergic neurodegeneration. To assess if our analyses are of translational value, we next applied the newly developed SEM analysis on a set of data from human PD patients treated with STN-DBS who had EEG recordings in the MCx, STN LFP analyses using an Active PC + S implantable pulse generator and DaTScan uptake analysis of the caudate nucleus and the putamen. We successfully defined two SEM models to analyse the relationships between β activity and clinical motor scores in human PD patients. In the first model, high β power and burst amplitude from the MCx, with corresponding parameters from the STN as a mediator, emerged as strong predictors of the UPDRS III motor Med-OFF score. In the second model, low β power from both the MCx and STN, combined with striatal dopamine uptake from the caudate nucleus and putamen as a mediator, showed a strong association with the same clinical score.

Our approach holds potential translational significance, especially when evaluating the neuroprotective impact of STN-DBS in PD. While the neuroprotective effectiveness of STN-DBS has been demonstrated in various rodent models of PD, the ethical and methodological concerns have thus far prohibited clinical trials in PD patients.^15,48^

There are limitations to the study, given that it was primarily built on preclinical data. No animal model fully replicates PD pathology, including the AAV-A53T-αSyn PD model, even though it has advantages over toxin models because of its αSyn-based pathophysiology. The challenge of modeling age-dependent PD progression remains due to rodents’ shorter lifespans, though longer observation periods offer some insights. Human brains are anatomically complex compared to rodents, with larger cortical regions and a more distinct separation of structures like the caudate and putamen. Rodents lack human-specific PD features like tremor, and gait studies highlight differences due to quadrupedal versus bipedal locomotion. Moreover, in our study we utilized the cylinder test to assess hemiparkinsonian behavioural deficits, opting not to perform additional behavioural tests to minimize stress for the rodents. Moreover, it is important to highlight that the patient cohort in this study comprises individuals with early-onset PD, which may constrain the generalizability of the findings—particularly those pertaining to β activity—to the broader population with idiopathic PD.

## Supplementary Material

awaf063_Supplementary_Data

## Data Availability

All data generated or analysed in this study are included in this article and supplementary files, or are available from the corresponding author upon reasonable request, except the data/results for human subjects that cannot be shared due to data protection.
