# Supplementary material for: Low β predicts motor output and cell degeneration in the A53T Parkinson’s disease rat model
Source: Brain. 2025 Feb 18;148(11):4058–71. doi: 10.1093/brain/awaf063 (PMC12588680; doi:10.1093/brain/awaf063)
Supplement: awaf063_Supplementary_Data [file awaf063_supplementary_data.pdf]

Table S1. Demographics and clinical scores for subject under investigation

Patient ID	Sex	Age	Disease Duration (Years)	UPDRS III (pre-op) stim-off/med off	UPDRS III (post-op) stim-on/med-off	LEDD (pre-op, post-op)
Wue02	M	65	10	40	19	1100, 800
Wue03	M	61	18	40	13	2725, 600
Wue04	M	54	7	26	23	658, 400
Wue06	M	51	11	47	9	1133, 180
Wue07	M	61	10	43	19	650, 220
Wue10	M	56	10	69	14	1200, 550
Wue11	F	53	11	55	6	1300, 460

UPDRS Unified Parkinson's Disease Rating Scale

LEDD Levodopa Equivalent Daily Dose

Table S2 Motor deficit correlations with low β parameters at week 8

Regions	LFP parameters	r-value	p-value
MCx	Power	-0.1818	0.5926
	Burst amplitude	0.1364	0.6893
	Long bursts (%)	-0.1364	0.6893
	Burst rate	-0.0459	0.8935
STN	Power	0.0979	0.7621
	Burst amplitude	-0.3566	0.2551
	Long bursts (%)	-0.3636	0.2453
	Burst rate	0.2697	0.3966

Table S3 Number of TH^+ SNpc neurons correlations with low β parameters			
**Regions**	**LFP parameters**	**r-value**	**p-value**
MCx	Power	-0.1182	0.7293
	Burst amplitude	0.0091	0.9788
	Long bursts (%)	-0.0636	0.8525
	Burst rate	-0.2385	0.4799
STN	Power	0.1958	0.5419
	Burst amplitude	0.3846	0.2170
	Long bursts (%)	0.4755	0.1182
	Burst rate	-0.0595	0.8542

Table S4 Striatal dopaminergic fiber density correlations with low β parameters			
**Regions**	**LFP parameters**	**r-value**	**p-value**
MCx	Power	-0.1273	0.7092
	Burst amplitude	0.1000	0.7699
	Long bursts (%)	0.2000	0.5554
	Burst rate	-0.1376	0.6866
STN	Power	-0.0769	0.8122
	Burst amplitude	0.2378	0.4568
	Long bursts (%)	0.3077	0.3306
	Burst rate	-0.2417	0.4492

Table S1 Demographics and clinical scores for subject under investigation

Table S2-4 Correlation analyses of low β (13-20 Hz) parameters with motor deficit, and nigrostriatal denervations at week 8.

Table S2 shows the correlations of low β parameters with motor deficit. **Table S3-4** represents the correlations of MCx or STN low β parameters with TH^+ SNpc neuron number or striatal dopaminergic fiber density. All r-values and p-values are summarized. *p <0.05, **p <0.01, ***p <0.001 and ****p <0.0001 for correlation analyses.

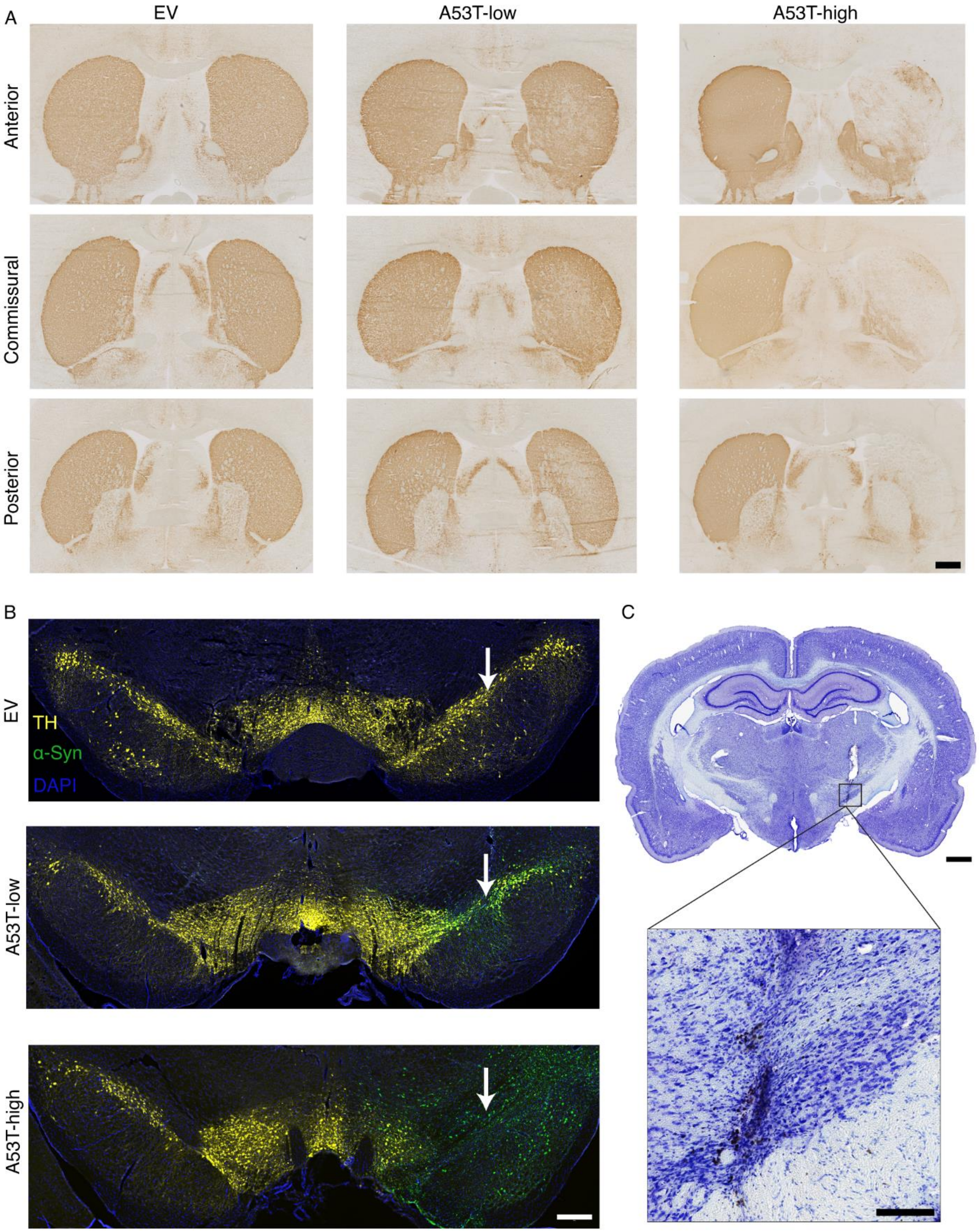

A
EV
A53T-low
A53T-high
Anterior
Commissural
Posterior
B
C
EV
TH
α-Syn
DAPI
A53T-low
A53T-high

Supplemental figure 1 (Fig. S1) shows histological evaluation of neurodegeneration in the dopaminergic nigrostriatal tract and verification of electrode placement in the STN. Representative images of TH^+ immunostaining in the anterior (first row), commissural (middle row) and posterior (bottom row) striatum (A; Scale bar 200 µm) used for optical density analysis in EV (first column), A53T low (second column) and A53T high (third column). Represenative images of immunofluorescence staining of TH (yellow), αSyn (green) and DAPI (blue) in EV, A53T-low and A53T-high; (B; scale bar 400 µm), Nissl-stained 40-µm coronal section showing the insertion track of the unilateral LFP wire electrode in to the right hemisphere (C; Scale bar 1000 µm), along with an higher magnification of the STN area with localization of the electrode tip. (C; Scale bar 200 µm);

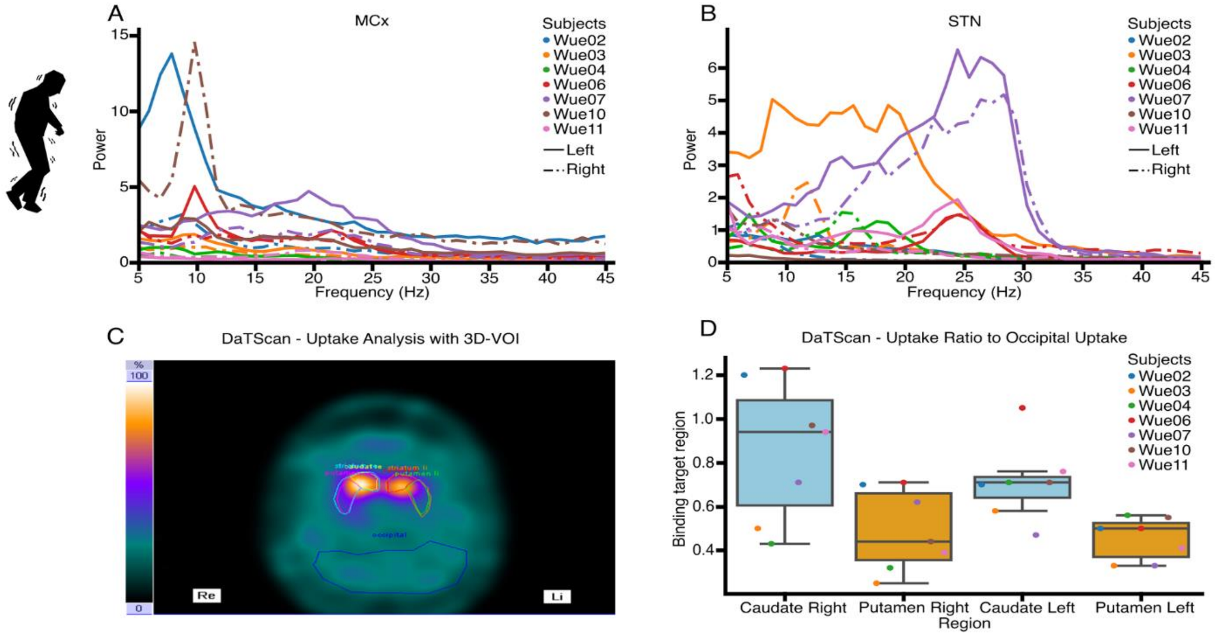

Supplemental figure 2 (**Fig. S2**) shows the power spectrum (Hanning window) of EEG reconstruction in the MCx for both hemispheres from all human subjects (**A**). **B**. Power spectrum of STN LFPs using Active PC+S implantable pulse generator (IPG). **C**. DaTScan uptake analysis 3D-VOI with highlighted target regions. **D**. Ratio of dopamine uptake in the striatum (i.e., Caudate nucleus and Putamen) to uptake by the occipital lobe for both hemispheres.
